# Converted Gods: Lives and Travels of Asante *Abosom* and *Asuman* Figures

**DOI:** 10.1080/17432200.2025.2505321

**Published:** 2025-07-11

**Authors:** Marleen de Witte

**Affiliations:** Department of Philosophy and Religious Studies, Utrecht University, The Netherlands

**Keywords:** Asante spiritual artifacts, colonial heritage, Catholic mission, missionary collections, African art, museums, artifact conversion

## Abstract

The ethnographic collection of the Dutch Spiritans holds six Asante shrine figures, whose journey reflects entangled histories of colonialism, indigenous West African religions, missionary Christianity, and cultural heritage. Originating in the colonial Gold Coast, these *abosom* and *asuman* passed through the hands of West African spiritual entrepreneurs, colonial police, “tribal art” dealers, Catholic missionaries, and museum curators. Along the way, their meanings, values, and powers transformed and accumulated, shaped by different collecting logics, material assemblages, display regimes, and epistemological frameworks. This article explores these shifts, examining how spiritual assets were redefined as “fetishes,” “tribal art,” ethnographic specimens, and cultural heritage. The role of the Catholic mission in the 1960s in promoting “African art” as a category of collection and display is highlighted as both challenging and perpetuating colonial frameworks. The concept of “cumulative conversions” is proposed to understand the layers of significance and agency built up over these artifacts’ lifetimes as latent potentialities that can be activated or deactivated as they move into new contexts. Particularly salient is the tension between treating such figures as museum/heritage “objects” and as channels for active spirit forces, with implications for heritage restitution and their potential roles in contemporary Ghanaian society and diaspora.

Behind the recently closed doors of the Afrika Museum in the Dutch village of Berg en Dal, a group of six anthropomorphic woodcarvings labeled “Asante altar figures” await their future in their vitrine in the abandoned “Religion and Society” exhibition (Figure 1).^1^ They happen to find themselves in the collection of the Dutch Province of the Catholic Congregation of the Holy Spirit. These “Spiritans” had long collected cultural artifacts, many related to indigenous religious and spiritual practices, from their mission fields in Africa and in 1954 founded the museum to display them permanently. The Asante figures did not arrive until 1967, however, when museum director Father Van Croonenburg purchased several “fetishes” from a Dutch dealer in “tribal art.”

**Fig 1:**
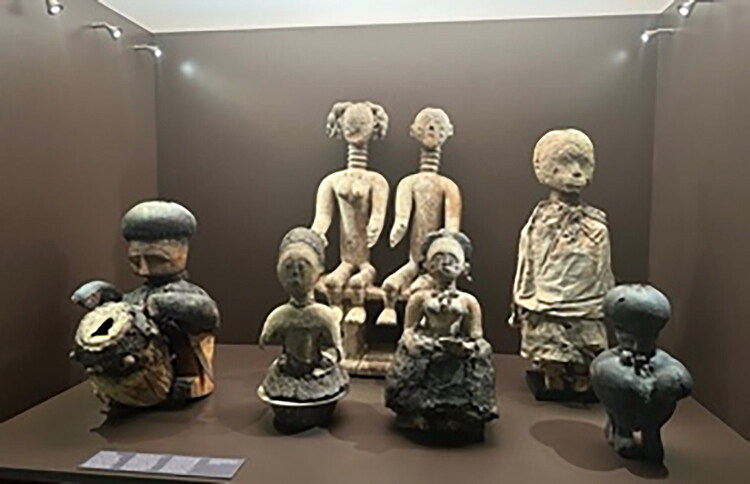
“Altar figures” (*altaarfiguren*) from Asante on display in the “Religion and Society” exhibition, 2023, Afrika Museum, Berg en Dal. Photo: Marleen de Witte.

Spiritual artifacts^2^ like these contain many interwoven histories of entanglement, displacement, and appropriation. More than a captive god in a museum informs us about the religion of an African community—“AM-104-1, power object, Asante, the god Tjuju, who mediates between man and supreme being” (Figure 1, second right)—it can tell us about the missionaries who collected and displayed it, the dealers who traded it, the museum visitors who came to look at it, and the ethnologists who studied it. It could also tell of activists who claim it as stolen heritage, artists who draw inspiration from it, and Pentecostals who would rather burn it. And, it can tell of the shrine priest who would ask AM-104-1 what it wants. Above all, it can tell of the connections and inequalities among all these people.

Missionary collections form a substantial, but often overlooked category of colonial heritage, comprising cultural artifacts (as well as natural objects) whose trajectories from their places of origin to their present locations are often far from straightforward (Jacobs, Knowles, and Wingfield 2015). Such multilayered, polyvalent and contested materials—caught up as they are in conflicting desires, interpretations, and valuations—offer an excellent entry point into the complex entanglements of colonialism/coloniality, missionary Christianity, West African indigenous religions, and cultural heritage. This article examines the lives and travels of the Asante *abosom* (gods) and *asuman* (spirits/deities) in the Spiritan collection, elucidating the various contexts and ways in which they were collected, used, described, and displayed, and the multiple meanings, values and powers they thus accumulated. In tracing their trajectories of transfer and transformation, I am interested in what these artifacts’ life stories might tell us about religious encounters and entwinements in the larger histories of colonial and postcolonial entanglement of Africa and Europe; and about how such religious materials might help address and redress the colonial past and its afterlives.

## Artifact Biographies and the Question of Conversion

Taking artifacts in motion as entry points into larger sociocultural and historical worlds, I draw inspiration from Igor Kopytoff’s “object biography” approach (Kopytoff 1986) to explore the various social lives (Appadurai 1986) that artifacts develop during their travels and life stages. I also build on more recent theorizing about “objects” or “things” as having some form of agency, as well as on West African knowledge traditions that have long produced similar ideas and expressed these through aesthetic practices. In particular, the notion that the agency of objects lies in the reciprocal, dynamic interaction between objects and humans (Saurma-Jeltsch 2010: 15; Basu 2017: 3) echoes the idea in many West African traditions that what makes certain objects powerful and agentic is humans’ sustained material and emotional engagement with them (an idea long scorned by European misunderstanders as “fetishism”) (Kedzierska Manzon 2013). By engaging the body and the senses, material artifacts connect people to larger worlds, and to the forces that animate them, on corporeal and affective levels. In the performative contexts of their social lives, artifacts *do* things, *act* on people, *set* things in motion, and *produce* identities and relations. Artifacts are not just material things made by humans; artifacts themselves make humans and constitute worlds. Approaching the nexus of religion and coloniality through this worldmaking power of artifacts, I ask what happens to this power when spiritual artifacts are displaced and appropriated in new (missionary and secular) contexts and interactions.

Over the course of their lives and travels from the colonial Gold Coast to the Netherlands, subsequent collecting logics, material assemblages, display regimes, and epistemological frameworks radically altered what these *abosom* and *asuman* were and did to the humans around them—as potent gods and spirits, as “idols” and “fetishes,” as “tribal art,” as “ethnographic objects,” as “cultural heritage.” In our project *Heritage and the Question of Conversion* (see introduction to this issue), we look at such trajectories and transformations through the lens of conversion, considering both the Christian project of conversion at the base of missionary collecting *and* the conversion of the artifacts thus collected into new kinds of objects, or indeed into “objects.”^3^ As an anthropologist interested in lived religion, I understand conversion, first, as not only a religious process. In a colonial context marked by strong hierarchies and inequalities, conversion to Christianity meant access to education, social mobility, political power, and modern life (Van der Veer 1996). The conversion of artifacts, too, is driven by colonial and postcolonial power imbalances. Second, I see conversion as not just of the mind—a question of belief or inner conviction—but as much as a matter of the body and the senses (De Witte 2011). Likewise, converting artifacts entails not just new meanings, but also new material practices. What is done (and no longer done) to the artifact’s “body” affects what it can do in each context, and how it relates to humans and to spirits. Third, while Christian doctrines generally see conversion as a linear progression from one state to another, in practice it involves back-and-forth movement and multiple, coexisting religious affiliations and practices. I suggest that the ongoing conversion of artifacts, and therewith of the relationships they mediate, is similarly messy: not a change from one sort of thing into another—*ɔbosom* into mission trophy into artwork into ethnographic object into economic asset into heritage—but a palimpsestic accumulation of layers (cf. Ames 1992: 141) that may be activated or deactivated as an artifact moves into new contexts. This has implications for thinking about their future potentialities, especially in the context of the debate on restitution and the engagement of contemporary Ghanaians.

## Asante Shrine Figures in the Afrika Museum

The Afrika Museum in Berg en Dal was founded in 1954 as a mission museum by the Dutch Province of the Congregation of the Holy Spirit. Grown out of the collecting activities of the Dutch Spiritans in their various mission fields in Africa, the new museum, established by missionary Piet Bukkems, was to inform the Dutch public about the importance of the mission in Africa in order to raise support and recruit young men for the mission. More generally, it was to offer the public an insight into the lifeworlds of the people they lived and worked among. While a discussion of the history of the Afrika Museum is beyond the scope of this article (see Eisenburger 1988; Welling 2002; Pels 2023), the museum has seen several transformations over the years, related to changing societal perspectives on Africa and on missionary work. With its 2014 incorporation into the umbrella organization of the National Museum of World Cultures (NMVW), the religious angle of the former mission museum was, according to the NMVW website, definitively left behind. However, as I will discuss below, the museum’s transformation into an ethnographic museum had already begun in the late 1950s. At the same time, its missionary legacy remained, most clearly on the upper floor, where until the closure in November 2023 the exhibition “Religion & Society” presented the old Spiritan collection of African spiritual and religious artifacts.

Upon entering the darkly lit exhibition space, one soon encountered, behind a glass window across from an introductory wall text on “Traditional Religions,” a set of “altar figures” (*altaarfiguren*) from Asante (Figure 1). The largest of them—about 70 centimeters high—stood in the center: a couple, a woman and a man, sitting on one stool. They were surrounded by other figures: a fairly large one dressed in white cloth, its face covered in white clay; two smaller, female figures in front, one placed in a brass bowl, her arms broken off, and one seated with her hands in her lap, both sitting in heaps of organic material; a drummer figure on one side and a figure covered in thick layers of black material on the other, both carrying metal knives at their waists.^4^ The label told us what there was to know about them: the seated couple are “possibly an ancestral queen and king,” the other figures “represent deities mediating between man and the supreme being.” Much remained unclear about their identities, however. Nor did we learn the Asante terms for such figures: *nsamanfoɔ*—ancestors; *ɔbosom* (pl. *abosom*)—a term denoting both deity and its material manifestation through which it can interact with humans; and *suman* (pl. *asuman*)—a different and broad category of artifacts imbued with spiritual power, ranging from amulets worn on the body to statues and other artifacts kept and cared for in secluded shrine rooms.^5^ Here they stood exposed behind their window, and random people would come up to gaze at them and snap their pictures.

My first encounter was with one of them—the white-clad figure—on the NMVW collection website, as an individual “power figure” (*krachtbeeld).* I was intrigued by its appearance and its asserted identity, “the god Tjuju,” a name I had never heard in my research on indigenous religion in Ghana.^6^ But what intrigued me most was the note that it “allegedly originated from a royal shrine” (an origin, I later learned, attributed to the entire group). If these figures were once part of a “royal shrine,” how did they end up in this museum? What happened to that royal shrine? Did a chief or priest convert to Christianity? If so, why? When I later visited the exhibition with a group of Netherlands-based Ghanaians, they wondered the same.^7^ Astonished to see such *abosom* here—certainly not objects to be gifted or sold; and not objects they would often (if ever) see up close—they asked: How come? What happened? The exhibition did not answer that question. In the ethnographic tradition of exhibiting the non-Western Other, the focus was on the belief systems and religious traditions of Africa’s different ethnic groups, on explaining the objects in their “original context.” Convinced that a focus on their journeys rather than their origins can reveal complex colonial and missionary histories and entanglements, in what follows I attempt to reconstruct what (might have) happened, sketching the various phases and transitions in their lives and unpacking the collecting logics, material assemblages, display regimes, and epistemological frameworks imposed on them.

## Collecting and Converting *Abosom* and *Asuman*: Historical Trajectories

The collecting histories of the artifacts that ended up in missionary collections did not always begin with the intervention of European missionaries, or other European collectors, in their lives. The artifact-centered (rather than missionary-centered) approach I take implies that we consider (religious) collecting networks and practices in West Africa prior to European collecting. This brings to light the role of indigenous religious functionaries as collectors and the role of shrines, similar to museums, as places of collection and conservation.

### Collecting Powers from Nzema

**Fig 2:**
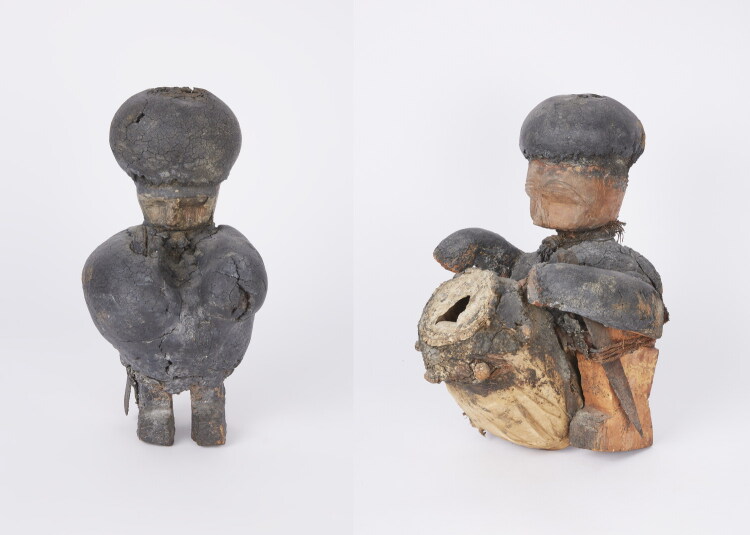
Collection Dutch Province of the Congregation of the Holy Spirit (CSSp), nrs. AM-104-3 “power figure” (*krachtbeeld*, earlier: “warrior fetish”) and AM-104-2 “figure” (beeld, earlier: “drummer fetish”). Photo: Esther van den Brink for NMVW.

In the museum’s collection database, we learn little about the historical context of the shrine figures. The old inventory cards indicate as their “origin” Konongo, a town not far from the Asante capital of Kumasi, but no time period. Comparing them to Asante carvings in other European collections, I found that the drummer and the warrior among them (Figure 2) were probably first collected by a shrine dedicated to a witch-catching deity in the early 1920s, called *Hwemeso*—literally “look after me.” They bear a strong stylistic resemblance to a wooden figure in the British Museum, documented as having been taken from an anti-witchcraft shrine in the 1920s.^8^ In his book *The Asante* (1981, 70–71), British Museum curator Malcolm Mcleod describes a broader class of Asante carvings to which the said figure belongs: often heavily coated in layers or lumps of “medicine” (*dufa,* potent organic material), these small, anthropomorphic wood-carvings, most of them coarsely carved and some with knives stuck into them, manifested the spiritual helpers (*boafo*) of powerful *asuman* imported from outside Asante. In the database of the African Heritage Documentation and Research Center, we find four very similar carvings, attributed to *Hwemeso* shrines around Konongo.^9^

The first decades of the twentieth century were a time of great unrest and tension, related to the subjugation of Asante to British colonial rule. These tensions were understood in terms of witchcraft and people felt the need to protect themselves, but their local *abosom* could not help—Parker mentions the belief that the Asante’s own *abosom* were too “lofty” to be of much use in the grim quotidian battle against maleficent witchcraft (2004: 401). New, powerful *asuman* deities were brought in from elsewhere through networks of itinerant spiritual entrepreneurs (Allman and Parker 2005; McCaskie 2004; Parker 2004) and their cults flourished. Between the 1900s and the 1950s numerous witch-catching cults followed one another. Many of these new deities, including *Aberewa*, *Nana Tongo*, and *Tigare,* came from the savannah areas to the North, but *Hwemeso* was traded from Apollonia (Nzema land) on the coastal border with Cote d’Ivoire, by one entrepreneur called Kwabena Nzima. It was valued for its effective problem-solving power, used for community protection, litigation and conflict resolution. The cult spread rapidly throughout Asante and Southern Gold Coast, including the town of Konongo (McCaskie 1981: 141). During my fieldwork in Ghana in September 2023, elders at the Konongo palace were amazed when I mentioned *Hwemeso* and showed me the site of its long-lost shrine, just behind the palace.

**Fig 3:**
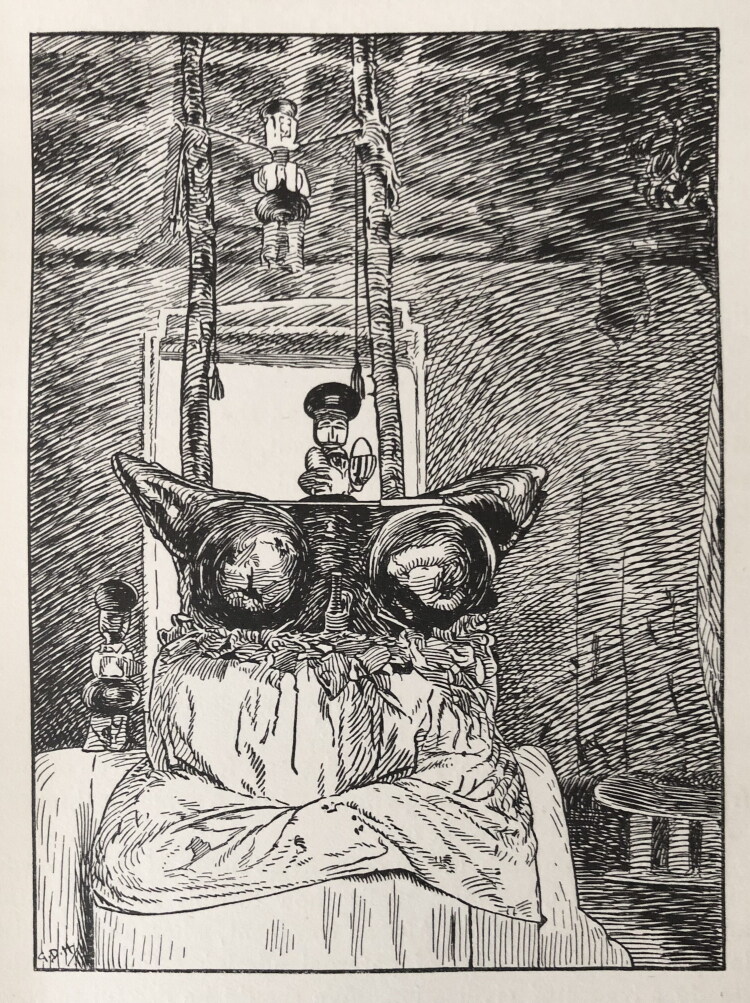
Sketch (made from photograph) by R.S. Rattray, “within the Fwemso witch-finders’ temple,” published in Rattray (1927, 33).

A drawing by the British colonial anthropologist Captain R. S. Rattray (1927, 33) (Figure 3) shows the larger material assemblage within which such carvings were arranged inside a *Hwemeso* (*Fwemso*) shrine (at Lake Bosumtwi, not far from Konongo)—as spiritual helpers to the *Hwemeso* deity, which itself consisted of two lumps of powerful material, sat on a raised altar covered with a white cloth, and was powered with (medicinal) leaves and several ritual knives. Such an *abosomdan* (room of the gods) would be accessible only to shrine attendants, and occasionally supplicants. Entry was subject to strict taboos and ritual requirements, such as bare feet—common to most shrines—and no sexual intercourse on Fridays and Sundays—specific to *Hwemeso* (ibid.: 32). It was a sacred space where people could interact with the gods and spirits, attend to their needs and invoke their help in return. This would include feeding them with raw eggs—I found tiny pieces of eggshell on the Afrika Museum figures—their favorite drink, and other offerings. This was not so much a visual display as an interactive installation designed to bring spirits into action. Rattray’s informant described how these helper spirits—with individual names, genders, and tasks—would transform into witch-like spirits themselves, to attract real witches, and wound or kill them with their knives (ibid.).

**Fig 4:**
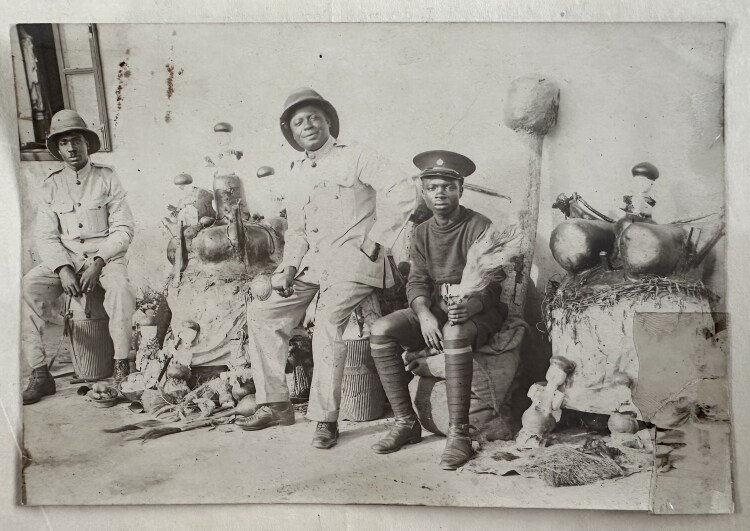
Colonial police with confiscated materials from Hwemeso shrine at Kwanyako, Gold Coast Colony, 1922. Public Records and Archives Administration Department Ghana, Accra, file nr. NAG ADM 11/1/1243, “Hwemisu Fetish.”

Its perceived effectiveness made *Hwemeso* very popular. Christian missions saw their recent converts “backsliding into heathenism” and fiercely combatted it. Witch-catching cults were also heavily suppressed by the colonial regime, that saw them as a threat to colonial order. The national archives in Accra keep a large folder with colonial documentation on the “Hwemisu Fetish” and its suppression (1922–1923). It includes two photographs of police confiscating *Hwemeso* shrine contents at Kwanyako (Figure 4)—used by the colonial regime as visual evidence to criminalize the cult. They show carvings like those in the Afrika Museum, amidst the larger assemblage in which they functioned, including the *Hwemeso* deity itself. The drums in the photos evoke the sonic dimension of this spiritual-material assemblage, as well as their silencing and disempowerment as part of the colonial eradication campaign.

Konongo elders did not know whether the *Hwemeso* shrine there had also fallen prey to police confiscation, nor what happened to confiscated items. It could well be, however, that this is how they ended up in the hands of traders, who then grouped them together with *abosom* of completely different pedigrees and characters.^10^ Shrine priests and other indigenous knowledge holders with whom I spoke were clear that the figures must have come from different shrines. From the photographs I had brought, all immediately deduced that these were gods of strictly distinct categories that cannot be placed together.

Aside from casting doubt on the figures’ alleged origin in a single “royal shrine,” the history of the transregional trade in ritual power from other places to Asante points to shrines as places of collection. Here, artifacts were brought in, sometimes from far away, and converted, through the necessary rites, into a new role, with a particular status and function in the community, tailored to local needs. This problematizes static and ahistorical notions of provenance and ethnic identification that came to dominate the carvings’ later, European interpretations. Rather than a place of origin, the shrine is better understood as a place of transit, just as the museum today is a place of transit rather than their final station.

### Collecting “Idols” and “Fetishes” from Pagan Africa

When and how the seven *abosom* and *asuman* disappeared from their Asante shrines is still unknown. Conversations with priests and elders in the Konongo area yielded several scenarios, including the possibility that they had been stolen by locals who were well-aware of the money Europeans would pay for “African fetishes.” Whatever happened, when they arrived at the Spiritans, they arrived as “fetishes” (as we can see from their inventory cards). For the missionaries, artifacts connected to indigenous spiritual practices and beliefs had long been a special target for collecting.^11^ The missionary project involved preaching to the so-called “pagans” that the gods and spirits they worshipped were “false” or “diabolical.” When people converted to Christianity—for a variety of reasons that are inextricable from the broader context of colonial power—they were required to break with their old gods and spirit powers and hand over the materials associated with them. This showed their “true” conversion. Despised by European missionaries and African catechists as “idols” and “fetishes,” many of these artifacts were publicly burned, as a way of destroying their power.^12^ Others were simply discarded and left to decay. But such artifacts were also avidly collected by missionaries, whose congregations had new uses for them at home.

Sent to Europe, such materials found a new role in Catholic seminaries and mission houses, where prospective missionaries could learn about what awaited them and prepare for the ardent task ahead. Artifacts like *abosom* and *asuman* became teaching tools used to visualize the “problem” of African “idolatry” and “superstition” and make palpable the spiritual backwardness and fear in which “the poor pagans” lived. They were also displayed to much wider audiences at traveling missionary exhibitions during the first half of the twentieth century. To generate interest and funds for the mission, missionary congregations put on bewildering displays of artifacts and natural objects collected from the non-Western world and the colonized populations they sought to convert (Coombes 1997; Groten 2019; Jacobs, Knowles, and Wingfield 2015). These were regular, quite large, and popular events, and thus played an important role in producing and spreading an image of these places and peoples.^13^ Although so-called “idols” and “fetishes” may have formed a minority of the items on display, in their stories and publications missionaries singled them out as graphic illustrations of the “deluded” condition of “paganism” and the mission’s victory over it (see also Roussillon, this issue). Displayed as “dreadful, ugly, unsavoury, and even terrifying idols” (1920 Mission Action Exhibition report, cited in Welling 2002: 40) amidst stuffed exotic animals, primitive weapons and other exotica, African gods and spirits became “missionary trophies” as well as powerful propaganda items, attracting public interest with the spectacle and affective excitement of the uncanny. As such, they were made to play a big part in feeding the popular imagination of the “black,” “heathen” and “wild” Other, as pitted against the Christian, civilized, white Self (Groten 2019: 496), embodied by the figure of the missionary manning the exhibition stand.

Missionary collections thus formed part of the colonial “cultural archive” (Said 1993) of narratives and images through which Europe invented Africa (Mudimbe 1988; Coombes 1997) and Africans as essentially different from, and inferior to, Europeans. Appropriated African spiritual artifacts materialize the entanglement of religion and race, or more precisely, of missionary work and race-making in the colonial encounter. Within the framework of the “fetish,” hierarchies of value ascribed to material objects, and people’s ways with them, were deeply intertwined with a hierarchy of value ascribed to bodies. Not only in the African “frontier zones” of colonization and missionization (Chidester 1996), but also in Europe, where the “fetish” objects rescued from iconoclastic destruction came to partake in installing a mental and affective grammar of racial inequality (Wekker 2016) in early twentieth-century European populations. Whether or not the Asante *abosom* and *asuman* in the Spiritan collection ever performed at mission exhibitions, the broader history of these exhibitions and their impact on popular imaginaries of Africa has coated them with a layer of meaning and affect that—even as missionary renderings would shift in later years—still informs current attitudes toward such artifacts, both among white European and Ghanaian publics.

### Collecting “Tribal Art” from Black Africa

According to the museum’s documentation, the “Asante altar figures” were bought for the Afrika Museum in 1967 by the museum’s director and curator, Father Jan van Croonenburg, from a Dutch dealer in “tribal art,” Henri L. Schouten. So it is only at this point that “Dutch missionary collecting”—our research focus—intervenes in their lives. At least, as far as I know now; their documented trajectory does not go back further than their acquisition date. Online research on Henri L. Schouten suggests that he acquired many of his pieces from other European collectors, including British ones. He also had connections with various missionary congregations and, like other “tribal art” dealers (Corbey 2000), may have sourced pieces from old missionary collections. While it may be impossible to trace the *abosom* and *asuman*’s exact journey from Konongo to Berg en Dal, their route *via* the art market is significant in pointing to the close entanglements of Dutch missionary collecting and the international trade in African “tribal art.” Missionaries bought and sold on this market, both informally and in more formalized ways, thus participating in the conversion of (selected) spiritual artifacts into art commodities with aesthetic and market value.

When the Afrika Museum was founded in 1954, African ritual and religious artifacts were still displayed as representative of a “strange world of idols and sorcery” (Eisenburger 1988: 36), similar to the traveling mission exhibitions. But from the late 1950s the approach shifted to an aesthetic admiration of African religious artifacts as culturally vital works of art (see also Pels 2023: ch.7). Van Croonenburg was a driving force behind this shift. It was a time of decolonization, when African colonies were gaining independence, and of the Second Vatican Council (1962–1965), which spurred the issue of “inculturation.” In the spirit of the times, mission congregations revised their earlier views of African culture. Van Croonenburg, who had never been to Africa, valued African art objects for their power to speak of “the deeply rooted cosmic worldview behind the entire African culture” and to “testify to the core of the thinking of the Black African,” as he wrote in the 1971 exhibition catalogue *Living Past* (Van Croonenburg 1971 *Levend Verleden*, translations from Dutch MdW). His drive was to introduce the Dutch public to the “fascinating cultural life” of “the Black African,” and to replace the still prevailing “astonishment and derision of the Black African” with an understanding and appreciation of his life and thought. To expand and improve the museum’s collection, Van Croonenburg began buying from his personal network of dealers, including Henri Schouten. But while Schouten sold him the Asante shrine carvings as “fetishes,” Van Croonenburg used the language of art, culture, and religion. What transpires from his writings is the missionary motive of encountering and understanding the African Other as fellow human being (*medemens*). This was the updated “mission” the Spiritans gave to their collection, and the power they attributed to African religious art works.

**Fig 5:**
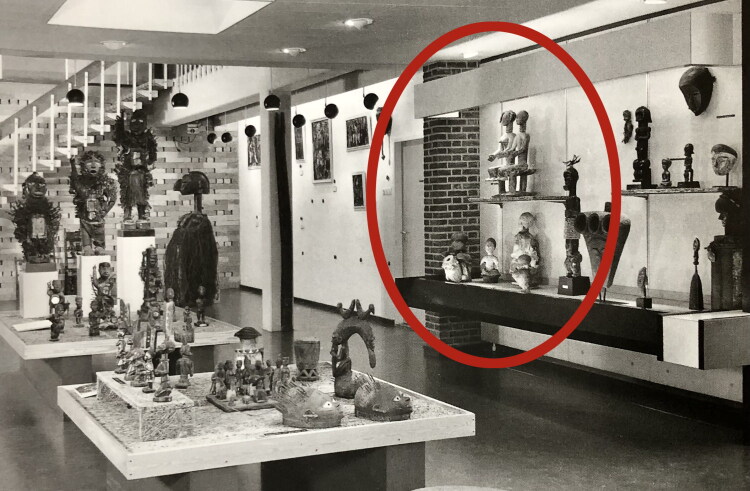
Open display of collection objects in the Afrika Museum, 1970s. Photo archive Afrika Museum, Berg en Dal, published in Welling (2002, 50).

A picture from the museum’s photo archive (Figure 5) shows how the shrine figures were arranged in the museum, with the “royal couple” on a shelf and the other figures below them. Unlike today, the artifacts were on open display, not behind glass, and with minimal textual information; nothing was to disturb the visitor’s aesthetic encounter with Africa. This was a purely visual encounter, however, cut off from the sounds, smells and tactile interactions of the figures’ shrine life. Exhibited as part of a collection of African art works, the *abosom* and *asuman* from Konongo became aestheticized objects for contemplative viewing, rather than the spiritual problem-solving workers they once were. In a way, coincident with their original role of “killing” evil spirits born of the vices of colonialism, Van Croonenburg also summoned them against the lingering spirit of colonial superiority. But the specific and often incompatible characters of the various spirits and deities that once worked through them, and their histories of migration, adaptation and suppression, were lost in their conversion to the Western category of art. As was the larger part of the material assemblages they were originally part of and functioning in—only deities and spirits who come in figurative form became “art”; and their drums and other accoutrements ended up in other categories, if collected at all.

The missionary shift to the art paradigm occurred in the broader context of growing European interest in the “primitive” art of non-Western peoples. This began in the early twentieth century, when modernist artists such as Picasso and many others found inspiration in African religious artifacts. Attracted by the “grotesque” expressiveness of African sculpture, they felt in it a powerful counterpart to their disillusionment with Western “civilization,” stemming not least from their abhorrence of the brutal colonial violence this “civilization” perpetrated in Africa (Leighten 1990). Appropriated to subvert Western aesthetic canons in the name of a “primordial authenticity,” African forms were soon widely celebrated for their artistic quality (Probst 2022). As a great hunt for African sculpture ensued, missionaries became suppliers of this art, selling or donating artifacts collected as “idols” and “fetishes” to private collectors, including artists. Over time, missionaries also came to value such artifacts as vital, genuinely inspired expressions of the totality of life, and became art collectors themselves. Critical of the art world’s purely formal appreciation of African sculpture, the Catholic missionaries of the Afrika Museum emphasized the spiritual quality of its aesthetic: the universal, all-encompassing “life-force” (*levenskracht*)^14^ of the great creator (*grote schepper*) that animates (*bezielt*) this art,” which, Van Croonenburg (n.d., 10) stressed, “to the African is not art” but “a form of religion.” By the time he bought the Asante figures for the museum in 1967, they had shifted categories from *asuman* and *abosom* to “fetishes” and “idols” to “art” (Leyten 2015). This development was less linear, however, than such a phrasing would suggest. Earlier layers of signification lingered on in this new chapter of Europe’s invention of Africa: the imagined primordial spiritism projected onto Africa and its art, the racialized Otherness, as well as the sense of the uncanny that such works continued to evoke.

**Fig 6:**
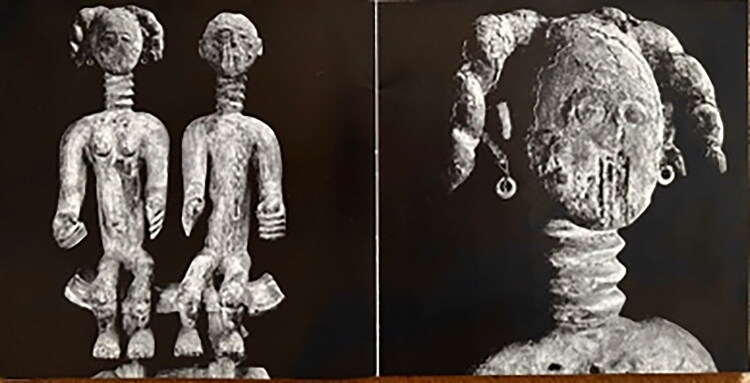
Photographs of AM-125-2 reproduced on page 4 and 5 of exhibition catalogue *Levend Verleden*, 1971.

Van Croonenburg must have ascribed a special affective power to the ancestral couple from Konongo, for their photos featured prominently in the exhibition catalog, with a close-up of the woman’s worn face (Figure 6). Much more can be said about the intention to make such artworks “speak” on behalf of “the Black African” (Pels 2023: ch.7), but let me note here the striking contrast to, first, the silenced voices of the African persons who made, used, and parted with these artifacts; and second, the silencing of the figures themselves, active beings in Asante thought and praxis. Turned into museum “objects,” they no longer speak the voices of gods and ancestors. As one Ghanaian visitor said as we stood in front of the vitrine some fifty years later, “They are here, but they don’t speak.”

### Collecting and (Not) Knowing “Ethnographic Objects”

From the outset, the Afrika Museum had academic ambitions as a place for ethnological and linguistic studies. This was reinforced by its increasing professionalization. With the ethnological value of the collection, the ordering and documentation of it became important, as did the desire for “ethnographic completeness.” Van Croonenburg thus sought to fill gaps in the collection through purchases and exchanges on the art market, targeting pieces from regions where the Spiritans were not missionizing, such as Asante. The *abosom* and *asuman*—sold as fetishes, acquired as art—were thus also given scientific value as objects for ethnographic study.

**Fig 7:**
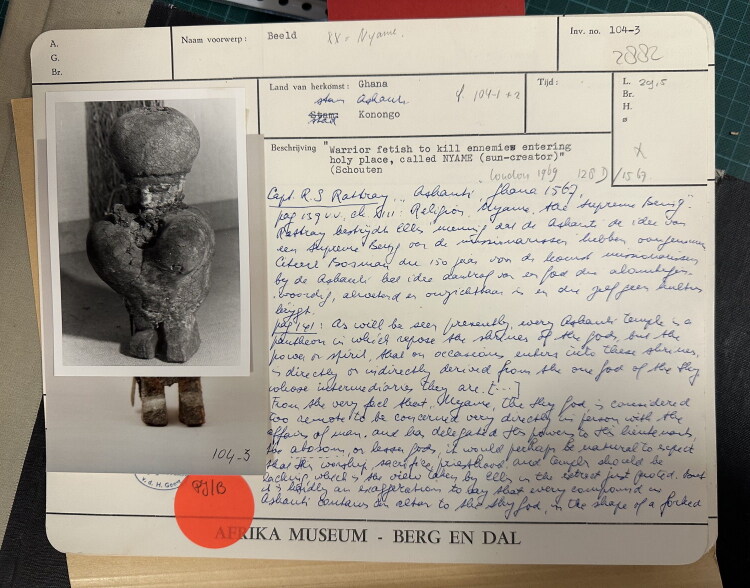
Inventory card of AM-104-3, Afrika Museum, Berg en Dal.

Examining their original inventory cards (Figure 7), we can see how, like captives, they were numbered, renamed, classified, described, their measurements and their “identity pictures” taken. Their origin was defined and registered: not attributed to an individual maker or a workshop, but to a “tribe” (*stam*).^15^ Their date of entry to the museum was registered, but no information was included about when they were made or used, nor when they were taken away. Finally, they were stamped “property of the Congregation of the Holy Spirit.” Such claims raise critical questions about property regimes and conflicting notions of ownership that are at the heart of current debates about colonial collections. In relation to the process of objectification alluded to here, we may note that *abosom* were never “property” in the first place. While some *abosom* and especially *asuman* could be “bought,” a priest/priestess’s relationship with them was not one of possessing them as material objects, but of attending to them as spiritual entities, who would in turn possess (*fa* – take) the priest or priestess as vessels and speak through them.^16^

**Fig 8:**
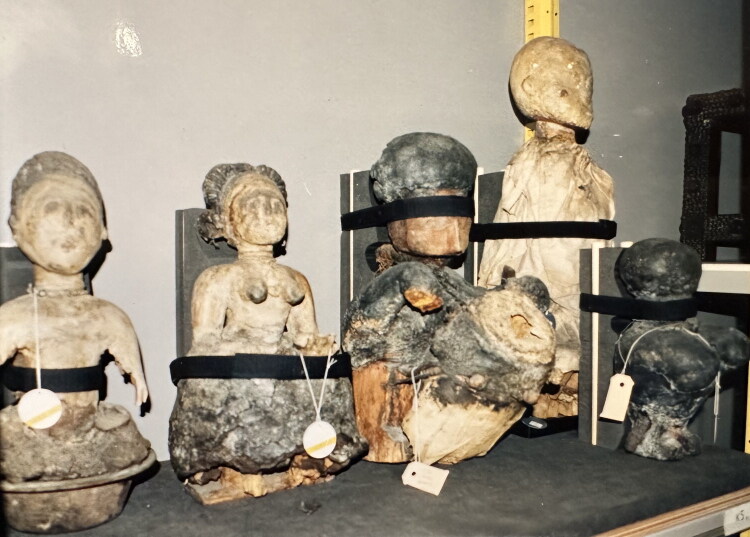
Members of the Konongo shrine group ready for transport to the Hague exhibit ‘Aspects: Akan cultures in Ghana’, 2001. Private photo archive Steven van de Raadt and Kathy van der Pas, reproduced with permission.

Turning *abosom* into “collection objects” is also a material process. As noted above, the conversion of artifacts involves not only new frames of meaning and evaluation, but also new material assemblages which alter their identity, their agency, and their relationship to humans and to spirits. In the museum, these assemblages include the other materials in the collection, but also the entire infrastructure of storage, preservation, transportation, and display (Figure 8); the evolving documentation systems, from paper cards in boxes and handwritten lists in bulky notebooks to the digital TMS system; the library of books and the study room; and the procedures for handling and treating everything classified as “collection.” From an artifact-biographical perspective, we see how the conservation regime, dedicated to preserving the material integrity of the “object” as it entered the museum, seeks to “freeze” it in time: from safety glass and latex gloves to pest control and climate and light regulation, great care is taken to prevent any material damage, decay or alteration. In the shrine, the figures received a very different form of care: they were fed with raw eggs and other substances and liquids, given a new dress for their annual festival, a new layer of “medicine” (*dufa*), or a new color of paint. They still bear the traces of such periodic renewal and “recharging.” In the museum, their material preservation as “objects” implied the spiritual starvation of the “living beings” they once were.

Handwritten notes on the inventory cards (Figure 7) give us a glimpse of how the *asuman* and *abosom* were studied as ethnographic specimens. The main interest was to understand the religious belief systems of different “tribes.” Documenting “the Asante belief system,” the museum curator at the time—and it may have been Van Croonenburg who wrote these notes, since in one instance they are dated 1974—turned to the work of R. S. Rattray. Stationed in Asante between 1911 and 1930, Captain Rattray was a British colonial anthropologist charged with the task of documenting Asante law, religion, customs, and art to assist the colonial administration in ruling the Asante. This was also valuable knowledge for missionaries (as Rattray himself suggested in his preface to *Ashanti Law and Constitution)*, who over time had developed an ethnographic interest in understanding indigenous African religions in order to “inculturate” the Christian message. And it was valuable knowledge for a museum that aimed to build and share knowledge about African cultures and lifeworlds.^17^ On the inventory card of the warrior *suman* (Figure 7) the curator copied passages from Rattray’s book *Ashanti* (1923) about the Asante idea of a Supreme Being, “the one God of the sky” (reflecting a particular missionary interest in and debate about Ur-monotheism) and the pantheon of “lesser gods” who served as his intermediaries. Practices of collection documentation thus turned the material manifestations of various spirit forces into objects of study, aiming to build ethnographic knowledge about the religion of the African Other, but distorting things in the process.

As African spiritual artifacts were subjected to the ahistorical ethnographic paradigm of the time, important historical knowledge was lost. In the Ghanaian spiritual landscape, deities have histories that are intimately linked to the histories of towns and communities (Warren and Brempong 1971). Shrines function as archives, where these histories are kept and transmitted (although less so today). When I discussed the Asante shrine figures with the Ghanaian historian Samuel Ntewusu, he immediately pointed to the warrior and the drummer: something serious must have happened in that community, he said, otherwise there would have been no need to bring in a warrior and a drummer spirit. In other words, these figures are not part of a timeless Asante pantheon of deities who mediate between humans and a supreme being; they were brought in and called upon in response to a particular historical event. This brings us back to the *Hwemeso* witch-catching shrines of the 1920s, with which, as I discovered after this conversation with Ntewusu, they were probably associated. Interestingly, the curator missed Rattray’s description of the new and “famous fetish Fwemso, which has lately been suppressed by Government” (1927, 31). Thus, while the ethnographic-missionary museum documented and presented such figures as ahistorical representative specimens of pre-Christian African religions—as outside European influence—they are in fact historical figures that emerged in response to and in interaction with colonialism and Christian missionization. If we want to know how such figures might help address and redress the colonial past and its afterlives, such knowledge may be crucial.

## Cumulative Conversions and Future Potentialities

There are still many unanswered questions about the lives and travels of the *abosom* and *asuman* from Konongo and the question of how and when they disappeared from their Asante homes and ended up on the international art market may never be resolved. But their journey to the Afrika Museum in Berg en Dal was evidently a tortuous one, passing through the hands of West African spiritual entrepreneurs, colonial police, “tribal art” dealers, Catholic missionaries, and secular museum staff. Along the way, different collecting logics, material assemblages, display regimes, and epistemological frameworks were imposed on them, affecting not only what they came to be and to do in the various contexts in which they were put to work. These multiple and conflicting impositions also had lasting consequences for their present and future potentialities.^18^

I propose that the ongoing conversion trajectory of the *abosom* and *asuman* not be understood in a linear way that annuls previous identities and powers. Rather, the various conversions they have undergone are better thought of as cumulative: layers built up over their lifetimes. Much like the layers of offering material on some of them, *abosom* and *asuman* accumulate latent potentialities that may be reactivated or deactivated (not nullified) as they move into new contexts. Their potential as spiritual problem-solving devices may have been deactivated by their “starvation” in the museum, as several Asante priests and elders pointed out, but could be activated again with the necessary rituals and offerings (De Witte and Meyer 2025). Their potential as commodities on the art market—deactivated by the museum regime, which declares the sale of collection items sacrilegious—could be reactivated if the Spiritan Fathers decided to sell them to raise money for their upkeep (as they recently did with an important *nkisi* and a Senufo carving in their collection). Even if, according to “tribal art” dealers I consulted, their market potential is limited by their “mediocre” aesthetic appeal, their status as economic asset is at stake in any negotiations about their future. Their agency as “fetishes” and “idols”—with undertones of fear, distance, and inferiority—may have been somewhat deactivated by the decolonial revisions of the 1960s. But, testifying to the power of Christianity to set enduring interpretive frames, it still works to alienate a young Ghanaian-Dutch audience confronted with the legacy of the “fetish” trope in influencing the images many Europeans have of Africans. And it resonates among Ghanaian Pentecostals, many of whom prefer to stay away from what they experience as socially stigmatized or spiritually dangerous things. But the Ghanaian delegation’s visits to the Afrika Museum (Furber 2022) also highlighted the potential of these artifacts as heritage items that can connect current generations of Ghanaians in the Netherlands to their ancestral culture.

It is precisely all these different layers of potentiality that make spiritual artifacts such as those biographed here so complex and ambiguous: cherished by some as cultural heritage, to others they may hold dangerous powers that warrant caution or even destruction. To assess the future potentialities of spiritual artifacts in missionary collections, it is crucial to recognize the multiplicity of their layers of meaning, value, and affect, and to explore the frictions as well as the points of connection between them. Particularly salient here is the tension between an objectifying—and, arguably, colonial—understanding of spiritual artifacts as museum/heritage/art “objects” and an understanding of them as potential channels for active spirit forces.

## Notes

1 In November 2023 the Afrika Museum closed its doors due to disagreements between the National Museum of World Cultures and the Congregation of the Holy Spirit.

2 Terms like “artifact,” “object,” “figure,” or “thing” do not do justice to the original ontological status of such material forms as agentic beings. Such terms – and qualifiers such as “religious,” “spiritual,” “African” or “Black” – need to be interrogated as part of examining the artifacts’ conversion trajectories.

3 Cf. Nick Thomas’ notion of the “converted artifact” (1991: 151).

4 Figure AM-125-4 was documented as male, but my interlocutors in Ghana identified its hairstyle as female. In September 2023 and January 2024, I carried out fieldwork in Ghana, including conversations around pictures of the shrine figures discussed here with people in indigenous religion, traditional authority, the Catholic Church, and the heritage field.

5 These categories of spiritual beings are rather fluid and may shift with time and context. Interpretations of these figures’ identities as *nsamanfoɔ, abosom*, or *asuman* varied among the indigenous religious specialists I spoke to, as did the distinctions between these categories.

6 No god named Tjuju is known in Ghana. The name is likely a misinterpretation of *juju*, an originally derogatory but also commonly used term for magical artifacts and practices.

7 Organized by Kate Furber as part of her MA research (Furber 2022).

8 BM registration number Af1938,0211.2.

9 The AHDRC online photo archive contains almost 220,000 entries of classical African art that have been auctioned, collected, published, exhibited, or documented otherwise. Object numbers ao-0168178, ao-0148391, ao-0051679, ao-0051680 are attributed to “Fwemso” and Konongo area by Doran Ross, Ghanaian arts scholar and Fowler Museum director and curator.

10 Space doesn’t permit me to discuss the backgrounds of the other figures, likely including the family of Tano river gods.

11 When the Afrika Museum was founded in 1954, first on the list of collectables was “everything relating to superstitious practices, witchcraft, and fetishism” (Welling 2002: 46).

12 “Fetish” and “idol” are terms with very different genealogies, but they have been used interchangeably in the West African mission field. On Christian missionary iconoclasm, see e.g., Corbey and Weener 2015; Strother 2016/2017; Meyer 2024.

13 Between 1919 and 1940, 171 mission exhibitions were held in the Netherlands, some attracting up to tens of thousands of visitors (Groten 2019).

14 A notion popularized by Belgian missionary Placide Tempels in his *Bantu Philosophy* (1945) and introduced to the Afrika Museum by Van Croonenburg’s predecessor Gerard Pubben (Pels 2023: 209ff).

15 Tribe (*stam*) and town (*stad*) were confused at the time of registration. Contrary to Asante concerns, tribe was found more relevant than town and Konongo was misregistered as “tribe.”

16 The Asante verb *kɔm* (to *kɔm* a god) denotes this reciprocal relationship of serving as a channel for and being possessed by a god.

17 Rattray’s work is still highly regarded in Asante today. His books are reprinted, and a park was named in his honor.

18 This is particularly pertinent given the precarious situation of the Spiritan collection (see note 1).

## References

[CIT0001] Allman, Jean Marie, and John Parker. 2005. *Tongnaab: The History of a West African God*. Bloomington: Indiana University Press.

[CIT0002] Ames, Michael. 1992. *Cannibal Tours and Glass Boxes: The Anthropology of Museums*. Vancouver: UBC Press.

[CIT0003] Appadurai, Arjun, ed. 1986. *The Social Life of Things: Commodities in Cultural Perspective*. Cambridge: Cambridge University Press.

[CIT0004] Basu, Paul, ed. 2017. *The Inbetweenness of Things: Materializing Mediation and Movement between Worlds*. New York: Bloomsbury Academic.

[CIT0005] Chidester, David. 1996. *Savage Systems: Colonialism and Comparative Religion in Southern Africa*. Charlottesville: University Press of Virginia.

[CIT0006] Coombes, Annie. 1997. *Reinventing Africa: Museums, Material Culture and Popular Imagination in Late Victorian and Edwardian England*. New Haven: Yale University Press.

[CIT0007] Corbey, Raymond. 2000. *Tribal Art Traffic: A Chronicle of Taste, Trade and Desire in Colonial and Post-Colonial Times*. Amsterdam: Royal Tropical Institute.

[CIT0008] Corbey, Raymond, and Karel Weener. 2015. “Collecting While Converting: Missionaries and Ethnographics.” *Journal of Art Historiography* 12: 1–14.

[CIT0009] De Witte, Marleen. 2011. “Touched by the Spirit: Converting the Senses in a Ghanaian Charismatic Church.” *Ethnos* 76 (4): 489–509. doi:10.1080/00141844.2011.620711

[CIT0010] De Witte, Marleen, and Birgit Meyer. 2025. “Consulting the Things of the Spirits: Evidencing Unseen Presences in Missionary Collections.” *Museum Anthropology* 48 (1). doi:10.1111/muan.70002.

[CIT0011] Eisenburger, Ineke. 1988. “Geschiedenis Afrika Museum.” In *Kunst Met Een Missie*, edited by J. Borger and J. A. M. Kerkhoff, 36–45. Maasbree: Werkgroep Musea-Missie-Medemens/Nederland Museumland.

[CIT0012] Furber, Kate. 2022. “‘So Much More than a Collection of Passive Material Things’: Investigating Contemporary Perceptions of the Congregation of the Holy Ghost’s Collection in the Afrika Museum, Berg En Dal.” MA thesis, Leiden University.

[CIT0013] Groten, Miel. 2019. “Difference between the Self and the Heathen: European Imperial Culture in Dutch Missionary Exhibitions, 1909–1957.” *The Journal of Imperial and Commonwealth History* 47 (3): 490–513. doi:10.1080/03086534.2018.1539725

[CIT0014] Jacobs, Karen, Chantal Knowles, and Chris Wingfield, eds. 2015. *Trophies, Relics and Curios? Missionary Heritage from Africa and the Pacific*. Leiden: Sidestone Press.

[CIT0015] Kedzierska Manzon, Agnes. 2013. “Humans and Things: Mande "Fetishes" as Subjects.” *Anthropological Quarterly* 86 (4): 1119–1151.

[CIT0016] Kopytoff, Igor. 1986. “The Cultural Biography of Things: Commoditization as Process.” In *The Social Life of Things*, edited by Arjun Appadurai, 64–91. New York: Cambridge University Press.

[CIT0017] Leighten, Patricia. 1990. “The White Peril and *L’Art Negre*: Picasso, Primitivism, and Anticolonialism.” *The Art Bulletin* 72 (4): 609–630. doi:10.2307/3045764

[CIT0018] Leyten, Harrie. 2015. *From Idol to Art: African ‘Objects-with-Power’: A Challenge for Missionaries, Anthropologists and Museum Curators*. Leiden: African Studies Centre.

[CIT0019] McCaskie, Tom. 1981. “Anti-Witchcraft Cults in Asante: An Essay in the Social History of an African People.” *History in Africa* 8: 125–154. doi:10.2307/3171512

[CIT0020] McCaskie, Tom. 2004. “Sakrobundi Ne Aberewa: Sie Kwaku the Witch-Finder in the Akan World.” *Transactions of the Historical Society of Ghana* 8: 82–135.

[CIT0021] McLeod, Malcolm. 1981. *The Asante*. London: British Museum.

[CIT0022] Meyer, Birgit. 2024. “‘Idols’ in the Museum: Legacies of Missionary Iconoclasm.” In *Reemerging Iconoclasms: On the Contemporariness of Image Controversies*, edited by Birgit Mersmann, Christiane Kruse, and Arnold Bartetzky, 108–130. Berlin: De Gruyter.

[CIT0023] Mudimbe, Y. V. 1988. *The Invention of Africa: Gnosis, Philosophy, and the Order of Knowledge*. Bloomington: Indiana University Press.

[CIT0024] Parker, John. 2004. “Witchcraft, anti-Witchcraft and Trans-Regional Ritual Innovation in Early Colonial Ghana: Sakrabundi and Aberewa, 1889-1910.” *The Journal of African History* 45 (3): 393–420. doi:10.1017/S002185370400951X

[CIT0025] Pels, Peter. 2023. *The Spirit of Matter: Modernity, Religion, and the Power of Objects*. Oxford: Berghahn Books.

[CIT0026] Probst, Peter. 2022. *What is African Art? A Short History*. Chicago: University of Chicago Press.

[CIT0027] Rattray, R. S. 1923. *Ashanti*. Oxford: Clarendon Press.

[CIT0028] Rattray, R. S. 1927. *Religion and Art in Ashanti*. Oxford: Clarendon Press.

[CIT0029] Said, Edward. 1993. *Culture and Imperialism*. New York: Knopf.

[CIT0030] Saurma-Jeltsch, Lieselotte E. 2010. “Introduction: About the Agency of Things, of Objects and Artefacts.” In *The Power of Things and the Flow of Cultural Transformations: Art and Culture between Europe and Asia*, edited by Lieselotte E. Saurma-Jeltsch and Eisenbeiß, Anja, 10–22. Berlin: Deutscher Kunstverlag.

[CIT0031] Strother, Zoe. 2016/2017. “‘Breaking Juju,’ Breaking Trade: Museums and the Culture of Iconoclasm in Southern Nigeria.” *Res: Journal of Anthropology and Aesthetics* 67-68: 21–41.

[CIT0032] Thomas, Nicholas. 1991. *Entangled Objects: Exchange, Material Culture, and Colonialism in the Pacific*. Cambridge, MA: Harvard University Press.

[CIT0033] Van Croonenburg, Jan. 1971. *Levend Verleden*. Berg en Dal: Afrika Museum.

[CIT0034] Van Croonenburg, Jan. n.d. *Met U Naar Afrika*. Berg en Dal: Afrika Museum.

[CIT0035] Van der Veer, Peter, ed. 1996. *Conversion to Modernities: The Globalization of Christianity*. New York: Routledge.

[CIT0036] Warren, D. M., and K. O. Brempong. 1971. *Techiman Traditional State, Part II: Histories of the Deities*. Legon: Institute of African Studies.

[CIT0037] Wekker, Gloria. 2016. *White Innocence: Paradoxes of Colonialism and Race*. Durham: Duke University Press.

[CIT0038] Welling, Wouter. 2002. “The Spiritans and the Afrika Museum: Changing Perceptions of Africa.” In *Forms of Wonderment: The History and Collections of the Afrika Museum*, edited by Jan-Lodewijk Grootaers and Ineke Eisenburger, 36–55. Berg en Dal: Afrika Museum.

